# Physical and Chemical Properties of Sintering Red Mud and Bayer Red Mud and the Implications for Beneficial Utilization

**DOI:** 10.3390/ma5101800

**Published:** 2012-10-09

**Authors:** Ping Wang, Dong-Yan Liu

**Affiliations:** 1 College of Civil Engineering, Chongqing University, Chongqing 400044, China; E-Mail: cquwangping@yahoo.com.cn; 2 Department of Military Architecture and Civil Engineering, Logistical Engineering University of PLA, Chongqing, 401331, China

**Keywords:** red mud, composition, radioactivity, hydraulic conductivity, application

## Abstract

Performances of two common types of red mud, Bayer red mud and Sintering red mud, were investigated in this research. Their compositions, mechanical properties and microstructure characterization were measured through XRD, TG and SEM analysis. Their shear strength, particle size, density and hydraulic characteristics also had been performed. Huge differences between the basic mineral types of these two kinds of red mud also can be found. The comparison of compositions shows that CaCO_3_ content in Sintering red mud is higher, Bayer red mud has more hazardous elements such as As, Pb and Hg and both have a high concentration of radioactivity. The micro particle of Bayer red mud is finer and more disperse, but the Sintering red mud has higher shear strength. Combining the TG and hydraulic characteristics analysis, it can be shown that Bayer red mud has higher value of water content and Sintering red mud has higher hydraulic conductivity. The paper then illustrates that Sintering red mud can become the main filling material of supporting structure of red mud stocking yard. Bayer red mud has a high reuse value and also can be used as a mixing material of masonry mortar.

## 1. Introduction

Red mud is the industrial waste generated during the production of alumina. According to the grade of raw material bauxite and the production process of alumina, red mud can be divided into Bayer red mud and Sintering red mud [1,2]. Based on present technologies, there is 0.8~1.76 t red mud generated by each 1t alumina produced [3]. It is reported that, there are up to 3 million tons of red mud produced by China’s largest three alumina production bases (Guizhou, Shandong and Henan) [4].

As there is a great deal of industrial alkali, fluoride, heavy metals and other potential pollutants in red mud, long-term stockpiling would not only occupy scarce land resources, but also easily lead to serious pollution of the surrounding soil, air and groundwater [5,6,7]. In addition, the continuous increasing of stockpiling yard height may lead to potential geological disasters [8]. Studies on the physical and chemical properties and comprehensive utilization of red mud have become a focus of related materials within science and engineering fields [9,10,11,12,13]. Through several measurements, Liu *et al.* [14] proved that pretreatment such as particle size classification of red mud is an effective way for the recycling of red mud. Paper [15] described the physical and chemical characteristics of red mud at different temperatures and provided a theoretical basis for the activation of red mud.

However, the different morphology and structure of Bayer and Sintering red mud determine the specific physical and chemical properties of red mud [16,17,18]. Therefore, it is important to distinguish the main characteristics such as chemical properties, mechanical performances, particle, morphology and structure for the comprehensive utilization of red mud [19].

In the present research, the characteristics of Sintering red mud and Bayer red mud have been measured through several routine analysis methods in the field of materials science and engineering such as XRD, SEM, TG, shear strength testing, particle size measurement and hydrodynamic characteristics testing. The paper then gives some suggestions for the comprehensive utilization of Bayer red mud and Sintering red mud separately.

## 2. Materials and Methods

### 2.1. Materials

**Figure 1:**
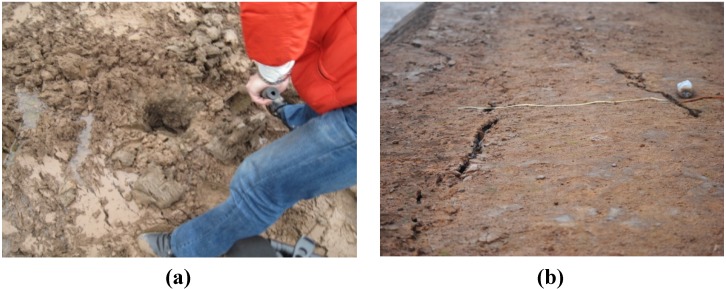
The field pictures of red mud: (**a**) red mud produced in Bayer processing (after pressure filtration); and (**b**) red mud produced in Sinter processing.

Both Sintering red mud and Bayer red mud were taken from an alumina refining plant in Guizhou, China. For the universality of the experiment results, each sample is a mixture of red mud stocked in six different sites for the same years (0.5 years). The stocking states of the two kinds of red mud are shown in Figure 1 (Bayer red mud often suffers an early pressure filtration progress before discharged).

The site maps show that, with the same stocking years, Bayer red mud has large water content (the value tested in laboratory is 52.3%) and is in a plastic-streaming state. While the Sintering red mud contains less water (the value in natural state is 37.5%), it shows a hard state and having white alkaline precipitation on the surface. Its strength measured by a Hand-held Concrete Rebound Hammer is close to C10 concrete.

### 2.2. Experimental Procedure

Samples were dried to constant weight at a temperature of 110 °C (fully dry, no free water) and ground to fine particles (<200 mesh).

The X-ray fluorescence (XRF) was performed on an XRF-1800X analyzer. The concentrations of several hazardous trace elements were detected by a NIOX300D inductively coupled plasma mass spectrometry (ICP-MS) produced by PerkinElmer (California, CA, USA). The activity concentrations measurement was performed on an AT6101C gamma-ray spectrometry system (ATOMTEX, Beijing, China). X-ray diffraction (XRD) analysis was carried out on a Rigaku (Tokyo, Japan) D/MAX 2500C diffractometer using GuKα radiation, voltage 40 kV, current 40 mA, and scanning angle of 5° to 75°.

Shear strength of the two kinds of red mud was tested on a DigiShear^TM^ (Trautwein, CA, USA) multi-functional direct shear testing systems with testing condition that: different normal stress (10 kPa, 20 kPa and 30 kPa) were loaded on the two samples separately, the shear rate is 0.03 mm/minute, the maximum shear displacement is 6.5 mm. These conditions can ensure the constant of water content of the samples throughout the tests.

The volume frequency of particle diameter is performed on a Winner2008A (WINNER INSTRUMENTS, Jinan, China) laser particle size analyzer, which has a measuring range of 0.05–2000 μm. The density measurement was carried out with a Model 1305 helium picnometer (Micromeritics, Georgia, GA, USA).

SEM observation was performed on TESCAN VEGA Ⅱscanning electron microscope to observe its 7500 times micro morphology of the two kinds of dried red mud. Thermal analysis was performed on a Netzsch (Germany) STA 449 simultaneous analyzer.

Thermogravimetric (TG) analyses were performed in the range of 25–625 °C (stripping gas: dry N_2_, heating rate: 15 °C/min).

The soil-water characteristic curve (SWCC) and hydraulic conductivity characteristics curve (HCF) was tested through a drying process on the two types of red mud by Unsaturated Soil Transient Cycle Test System (GDS UNSAT, Leeds, UK).

## 3. Results and Discussion

### 3.1. Chemical Properties

There are different aluminum production processes to different bauxites. Red mud is mainly composed of coarse sand and fine particle. Its composition, property and phase vary with the type of the bauxite and the alumina production process, and will change over time. The chemical composition of the two kinds of red mud determined by XRF analyzer is given in Table 1.

**Table 1:** The main chemical constituents of red mud (%).

Chemical constituent	Fe_2_O_3_	Al_2_O_3_	SiO_2_	CaO	Na_2_O	TiO_2_	K_2_O	Sc_2_O_3_	V_2_O_5_	Nb_2_O_5_	TREO	Loss
Bayer process	26.41	18.94	8.52	21.84	4.75	7.40	0.068	0.76	0.34	0.008	0.012	9.71
Sintering process	7.95	10.36	17.29	40.22	3.53	7.14	0.053	0.16	0.024	0.020	0.31	12.95

It can be seen in Table 1 that the main chemical compositions of red mud are Fe_2_O_3_, Al_2_O_3_, SiO_2_, CaO, Na_2_O, TiO, K_2_O and MgO. The CaO and SiO_2_ contents of Sintering red mud are much higher than that Bayer red mud, but its Fe_2_O_3_ content are low. In addition, Sintering red mud contains relatively more Rare Earth Elements than Bayer.

In addition to the major elements, the average concentrations of several hazardous trace elements including As, Pb, Hg, Cd and Cr were also detected by the ICP-MS analysis, Table 2. The Table 2 shows that the total concentrations of hazardous trace elements in Bayer red mud are larger than that of Sintering red mud. We then know that Sintering red mud has high content of hazardous trace elements.

**Table 2:** The concentrations of hazardous trace elements in red mud (ppm).

Hazardous trace elements	As	Pb	Hg	Cd	Cr	Ba	Zn	Cu	Mn	Ni	Total
Bayer process	267.3	56.6	67.3	27.1	537.8	212.0	103.2	78.2	187.5	984.9	2521.9
Sintering process	246.7	48.0	58.7	14.4	416.9	197.2	76.3	213.6	146.3	578.6	1996.7

Both the two have radioactive elements like ^226^Ra and ^232^Th, which may be another key problem of the further utilization of red mud. As to the measurements of radioactivity, V. Jobbágy *et al.* [20] have measured the activity concentrations of the natural radionuclides of red mud product in Hungary. The measured average (min–max) ^226^Ra and ^232^Th activity concentrations are separately 360 (150–700) Bq·kg^−1^ and 292 (102–506) Bq·kg^−1^, which is 2 and 14 times higher than the world average value of building materials. We also have performed measurement in the two kinds of red mud through a gamma-ray spectrometry system and the results are shown in Table 3. Compared with the sample from Hungary, our samples have relatively low concentration of ^226^Ra and high concentration of ^232^Th. As a consequence, it can be know that both Sintering red mud and Bayer red mud have high values of radioactivity.

**Table 3:** ^40^K, ^226^Ra and ^232^Th activity concentration of the samples.

**Red mud samples**	Average radionuclide concentrations (min–max) [Bq·kg^−1^]		
	^40^K	^226^Ra	^232^Th
Sintering red mud from Guizhou, China	87 (58–164)	276 (95–570)	389 (340–450)
Bayer red mud from Guizhou, China	113 (67–247)	302 (125–620)	404 (360–475)
Bayer red mud from Hungary [20]	48 (5–101)	360 (150–700)	292 (285–380)
World average of building materials [21]	500	50	50

**Figure 2:**
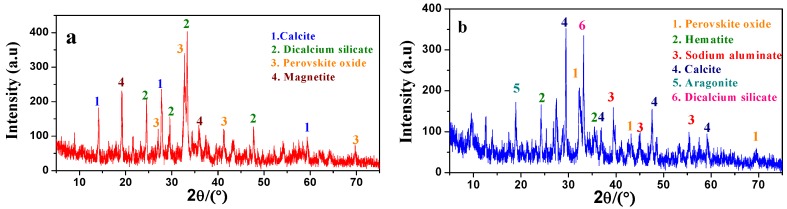
The X-ray diffraction of red mud: (**a**) Sintering red mud; and (**b**) Bayer red mud (after pressure filtration).

The XRD diagrams of the obtained Sintering red mud and Bayer red mud are shown as Figure 2. From the diagrams it can be known that the main mineral phases of Sintering red mud are Dicalcium Silicate (Ca_2_SiO_4_), Calcite (CaCO_3_), Perovskite (CaTiO_3_) and Magnetite (Fe_3_O_4_), *etc*. While, the main mineral phases in Bayer red mud are Perovskite (CaTiO_3_), Hematite (Fe_2_O_3_), Sodium Aluminate (Na_5_AlO_4_), Calcite (CaCO_3_) and the Aragonite (Ca(CO_3_)), *etc*. It is indicated that they have obviously different mineral compositions.

### 3.2. The Mechanical Properties

**Figure 3:**
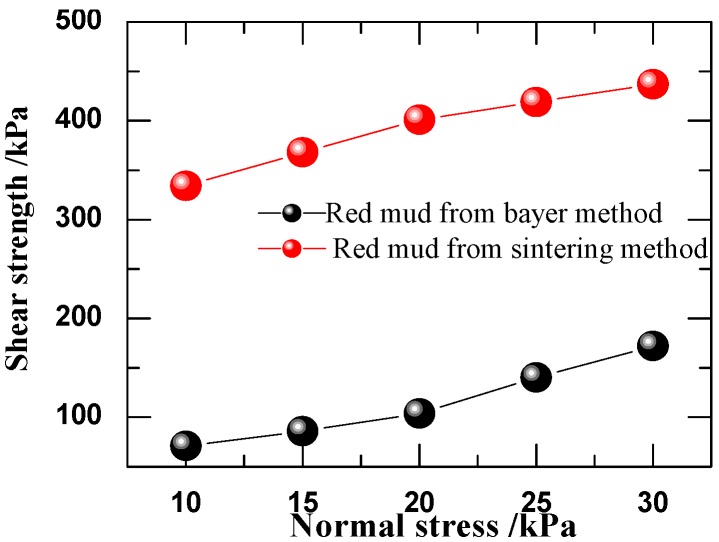
The direct shear test results of red mud.

The physical properties such as strength, particle size and density of Sintering red mud and Bayer red mud are different. The measurement on site shows that the strength of Sintering red mud is far higher than that of Bayer red mud under the same stocking years. Then the direct shear tests were carried out for the purpose of further understanding the strength performance of the two red muds. The testing results are shown in Figure 3.

The curves in the Figure 3 indicate that, under the same experimental conditions Sintering red mud has significantly high intensity. This is consistent with the conclusion obtained in the sites. The bond strength of the two samples can be calculated though extending the strength envelope curve towards left to the longitudinal axis of the coordinate system. The bond strength of Sintering red mud, 287 kPa, is significantly greater than that of Bayer method which is 14.6 kPa. In addition, it can be known that the angles of internal friction of the two materials are almost the same from the fact that two shear strength envelope curves are roughly parallel. These phenomena illustrate the main reason why the strength of Sintering red mud is substantially higher is come from its larger bond strength.

### 3.3. Particle Size and Density

**Figure 4:**
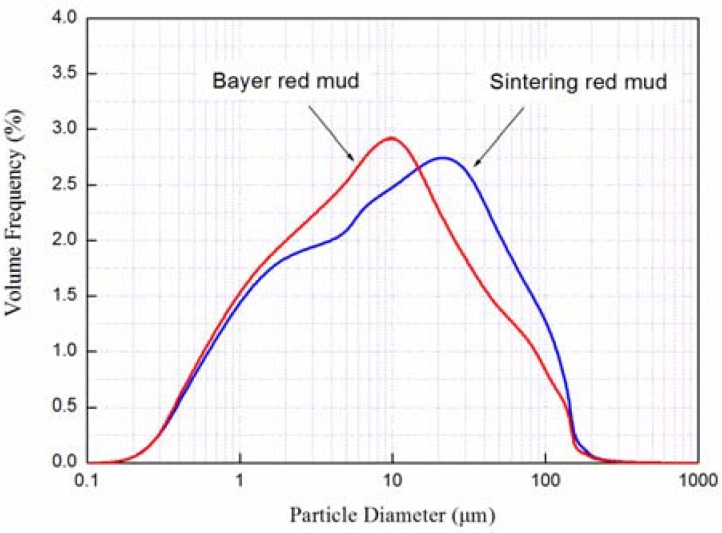
The particle diameter distribution of Sintering red mud and Bayer red mud.

The particles size distribution of the two kinds of red mud is shown in Figure 4. It can be seen that the ground Sintering red mud particles are mostly in the range of 0.7–100 μm with a mean value of 28.5 μm. Compared with Sintering red mud, the Bayer one has a relatively small particle diameter. The particle diameter of Bayer red mud is between 0.8 μm and 50 μm with an average value of 14.8 μm.

The measured values of density of red mud are 3.26 g/cm^3^ (Sintering red mud) and 2.70 g/cm^3^ (Bayer red mud). The differences of particle size and density values have significant influences on the performances of red mud as the raw materials of cement and concrete.

### 3.4. SEM Characterization

**Figure 5:**
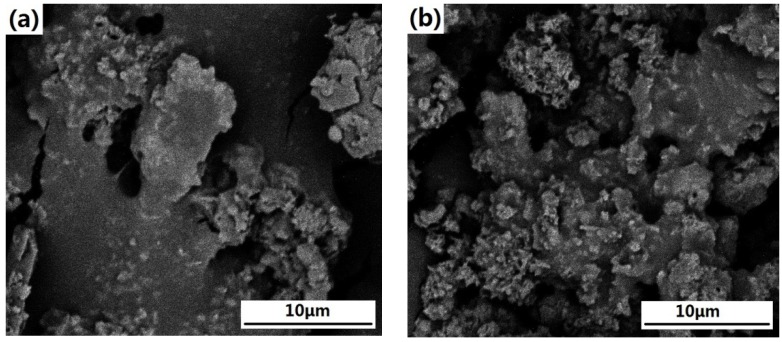
SEM diagrams of (**a**) Sintering red mud; and (**b**) Bayer red mud.

The SEM diagrams after chrominance processing are shown in Figure 5 (left for the Sintering red mud and right for the Bayer red mud). Combining with the conclusion obtained from the analysis of strength, particle diameter and density, SEM characterization is helpful for the further understanding of the physical performance and the microstructure of red mud.

A comparative analysis on the two images shows that the two kinds of red mud have relatively loose microstructures and high porosities. The particles of Sintering red mud (Figure 5a) are easy to gather into a cluster. On the contrary, Bayer red mud (Figure 5b) has more dispersive particles. In addition, the specific gravity of Bayer red mud determined by pycnometer method is 2.64, higher than that of Sintering red mud which is 2.47.

### 3.5. The TG Analysis

**Figure 6:**
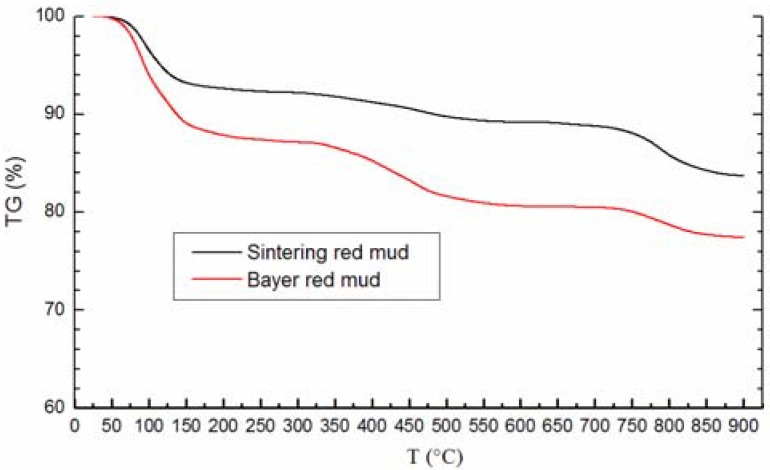
Thermogravimetric (TG) analysis diagram of red mud.

The TG analysis diagram (Figure 6.) of red mud is obtained by testing the mass loss through the heat treatment of red mud. In contrast, the TG curves of the two kinds of red mud have a different value of mass loss. It indicates that the Bayer red mud has a high value of water content than Sintering red mud.

Similarly, both the two kinds of red mud show a continuous weight loss from room temperature (25 °C) to 900 °C. On the other hand, both curves consist of three portions of mass loss progresses at different temperature interval. The first one is during the heating temperature interval of 25°C~150 °C. The mass loss (6.9% and 11.1%) is mainly caused by the evaporation of free water in the red mud at a low temperature.

The mass of the samples then undertakes a slow decline between 150 °C and 625 °C (3.9% and 8.3%). This proves the existence of hydrous minerals in red mud. It also indicates that the combination states and bonding strengths of water in the hydrous minerals are not the same, and water may exist in hydrous minerals with different states and combination strengths (such as crystal water and structural water). This feature is very similar to the state characters of present water in the C-S-H gel in Portland cement hydration products.

Above 625 °C, the curves have another decrease because of the decomposition of CaCO_3_. As the Figure 6 presents, from 625 °C to 900 °C, Sintering red mud lost 6.5% of its total mass, which is about one times more than Bayer red mud (3.2%). This result confirms the conclusion that Sintering red mud has more CaCO_3_ than Bayer red mud.

### 3.6. The Hydraulic Characteristics

**Figure 7:**
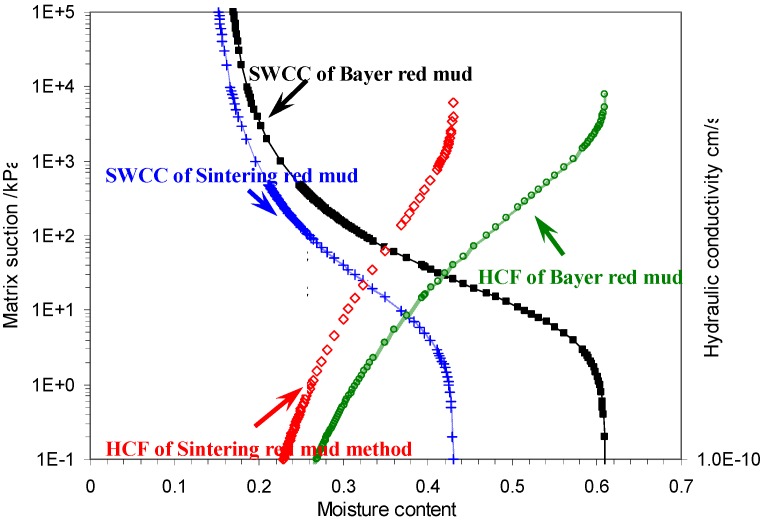
The hydraulic characteristics of two types of red mud.

Both Bayer red mud and Sintering red mud belong to alkaline industrial wastes. So the long-term stockpiling of alkaline red mud would not only occupy scarce land resources, but also easily lead to serious pollution of the surrounding soil, air and groundwater. Therefore, a deeply recognition of the hydrodynamic characteristics of red mud is necessary for the pollution prevention of red mud. The measured hydraulic characteristics can be illustrated by soil-water characteristic curve (SWCC) and hydraulic conductivity characteristics curve (HCF), which are shown in Figure 7.

The SWCC curves indicate that, with the same change of water content, the change of matrix suction of Bayer red mud (matrix suction value is a function of several factors like free water, electric combination water, cement force and electrochemical power) is greater than that of Sintering red mud. This means that the water sensitivity of Bayer red mud is greater than Sintering red mud. As a consequence, under the same natural conditions, the stability of Bayer red mud stock is lower than that of Sintering red mud. On the other hand, with the same water content, the hydraulic conductivity of the Sintering red mud is better. It indicates that the liquid pollutants in Sintering red mud are easier to filtrate and diffuse into the surrounding environment.

### 3.7. Applications of Red Mud

The microstructure characteristics analyses give a Comprehensive introduction to the chemical and physical properties of red mud. Compared with Bayer red mud, Sintering red mud has higher strength and larger density and particle diameter. This conclusion is helpful for the comprehensive utilization such as cement production and production of filling materials of red mud according to different types.

According to the TG analysis, the occurrence mode of water in red mud is very similar to that of water in C-S-H gel, which is one of the hydration products of Portland cement. Besides, the previous researches also proved that the fresh red mud contains some β-C_2_S and has good activity [22,23]. Thus, both the two kinds of red mud have the possible of large scale of application in the production of cement mixture.

#### 3.7.1. Sintering Red Mud

The comparison of compositions and XRD patterns of the two kinds of red mud shows that CaCO_3_ content in Sintering red mud is significantly higher than in Bayer red mud. So it will be more applicable for the production of cement. On the other hand, Bayer red mud has a high content of hazardous elements like As, Pb and Hg. Both the two kinds of red mud have a high concentration of radioactivity. So according to the study of Burke *et al.* [24], the influence of hazardous elements and radioactivity in red mud should be avoided when applying the red mud in the production of building material.

**Figure 8:**
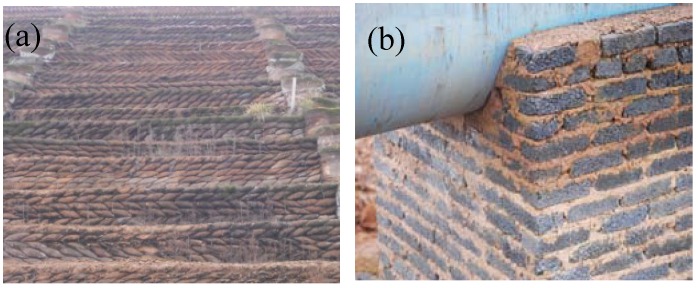
One of the comprehensive utilization method: (**a**) Sintering red mud used as the foundation; and (**b**) the catch drain.

Combining the bonding strength testing and hydraulic characteristics, it can be shown that under the same stocking conditions, the higher mechanical strength of the Sintering red mud, the more stable it will be. So it is applicable to be the main filling materials of the dam of stocking yard, which is shown in Figure 8a. But the high hydraulic conductivity of Sintering red mud will improve the infiltrate of alkaline solution. So it is indispensable to build a catch drain (as shown in Figure 6b) for the purpose of collecting the alkaline solution.

#### 3.7.2. Bayer Red Mud

Bayer red mud has a smaller particle size, lower density and larger surface area, and is more difficult to gather into a cluster. When added into masonry mortar, it can improve the porosity and water absorption rare and significantly enhance the performance of mortar. The present study also shows that the reason why the specific gravity of Bayer red mud is higher than Sintering red mud: it has a large proportion of metal elements. So Bayer red mud contains more value in the recovery of valuable elements, which has also been confirmed by the previous research results [25,26].

## 4. Conclusions

According to the above measurements and discussions, the paper presents a comprehensive illustration of the main chemical and physical properties of red mud and gives some suggestions about the comprehensive treatment of red mud. The main conclusions are as follows:

(1) The chemical and phase composition, mechanical properties, density and particle diameter, occurrence mode of water, hydraulic characteristics of the two kinds of red mud are different, which can determine the different chemical and physical properties.
(2) Both kinds of red mud have the possibility of large scale application in the production of cement mixture. However, the influence of hazardous elements and radioactivity in red mud should be avoided when applying the red mud in the production of building material.
(3) Sintering red mud, with its greater stability and hydraulic conductivity, can be the main filling material of supporting structure of red mud stocking yard. However, potential pollution of liquid pollutants to the surrounding environment should be prevented. Bayer red mud has a high reuse value and can also be used as a mixing material of masonry mortar.
